# Predictors of mHealth use in promoting adherence to pre-exposure prophylaxis among female sex workers: an evaluation of the *Jichunge* intervention in Dar es Salaam, Tanzania

**DOI:** 10.1186/s12913-022-08245-2

**Published:** 2022-07-04

**Authors:** Christopher Mbotwa, Method Kazaura, Kåre Moen, Melkizedeck Leshabari, Emmy Metta, Germana Leyna, Elia J. Mmbaga

**Affiliations:** 1 Department of Epidemiology and Biostatistics, Muhimbili University of Health and Allied Sciences, Dar es Salaam, Tanzania; 2 Mbeya College of Health and Allied Sciences, University of Dar es Salaam, Mbeya, Tanzania; 3 Department of Community Medicine and Global Health, Institute of Health and Society, Faculty of Medicine, University of Oslo, Oslo, Norway; 4 Department of Behavioural sciences, Muhimbili University of Health and Allied Sciences, Dar es Salaam, Tanzania; 5 Tanzania Food and Nutrition Centre, Dar es Salaam, Tanzania

**Keywords:** mHealth, *Jichunge*, Female sex workers, PrEP, HIV

## Abstract

**Background:**

There is evidence that pre-exposure prophylaxis (PrEP) is effective in preventing HIV transmission, and PrEP is recommended by the World Health organization (WHO) for use by individuals at high risk of HIV infection. However, low adherence has been reported to hamper its effectiveness. Some evidence indicates that mHealth interventions may be a promising way of promoting PrEP adherence. Nevertheless, evaluations of mHealth interventions in Africa, the region most affected by HIV, are scarce. This study aimed at identifying the extent of and predictors for use of a smartphone based mHealth application among female sex workers in Dar es Salaam, Tanzania.

**Methods:**

As part of a quasi-experimental study in Tanzania, 470 female sex workers who were eligible for PrEP and who owned a smartphone were recruited using respondent driven sampling. All participants were provided with an mHealth application called *Jichunge*, a smartphone-based app designed to promote adherence to PrEP by offering users information, advise and support during start-up and use of PrEP. We collected data through structured interviews at baseline and extracted user data from the app for a period of 30 days. Modified Poisson regression model with robust standard errors was used to identify predictors for the optimal use of the *Jichunge* app.

**Results:**

Overall, the optimal use of the *Jichunge* app was 46.4%. Optimal use was significantly higher among women who were older (aPR = 1.3, 95% CI: 1.10-1.65, *p* = 0.004 for age 25-34 years, and aPR = 1.6, 95% CI: 1.19-2.07, *p* = 0.001 for age at least 35 years), who had secondary education or higher (aPR = 1.8, 95% CI: 1.08-2.94, *p* = 0.023), who had suboptimal social support (aPR = 1.2, 95% CI: 1.02-1.48, *p* = 0.030), who had high awareness of PrEP (aPR = 1.3, 95% CI: 1.08-1.55, *p* = 0.005), and who had experience using common mainstream social media applications (aPR = 1.4, 95% CI: 1.08-1.71, *p* = 0.009).

**Conclusion:**

Optimal use of the *Jichunge* app was substantially higher among women with higher age, higher education, higher PrEP awareness, less social support, and experience using common social media applications. Individual and interpersonal factors should be considered in planning mHealth interventions. Further studies to determine predictors of longer-term mHealth engagement are needed.

**Trial registration:**

International Clinical Trials Registry Platform PACTR202003823226570; 04.03.2020.

## Background

Female sex workers, like members of other ‘key populations’, are disproportionally affected by HIV globally [1, 2]. In Tanzania, the HIV prevalence among female sex workers is more than two times higher than that of women in the general population [3–5]. Pre-exposure prophylaxis (PrEP) has recently been recommended by the World Health Organization (WHO) to be part of efforts to slow the rate of new HIV infection in groups at high risk of HIV, including female sex workers [6, 7]. Evidence indicates that the effectiveness of PrEP largely depends on users’ adherence to the medication regimen, which in Tanzania is a daily dose of a combination pill containing tenofovir and emtricitabine [8–10]. However, experience from antiretroviral therapy (ART) programming and a limited number of demonstration trials of PrEP suggests that low adherence to treatment may be a main challenge and that this could negatively affect PrEP effectiveness [11, 12]. This challenge calls for innovative interventions to promote adherence to PrEP.

The integration of mobile technology in the health sector (mHealth) has emerged as an effective way of promoting and delivering health care to different population groups [13–16]. mHealth applications have been shown to improve adherence to ART, and compliance with scheduled ART follow-up appointments, in many countries [17–19]. The use of mHealth can improve access to healthcare services for different groups irrespective of time and geographical location. Furthermore, mHealth applications appear to be a promising solution for promoting access to healthcare services especially in underserved populations, such as some HIV at-risk populations.

A limited number of studies, mostly from high-income countries, have demonstrated that mHealth may be a promising way of promoting uptake of, adherence to, and retention in PrEP services in groups of persons at elevated risk of HIV [13, 20–23]. For example, different text messaging interventions have been shown to significantly increase retention in PrEP care and adherence to PrEP among men who have sex with men in the United States [13, 20, 21]. Improving access to health services is especially important in populations experiencing discrimination and criminalization [24]. While mHealth seems to be promising, there is limited evidence on the use and evaluation of mHealth in the context of PrEP programming in at-risk populations for HIV in Africa, the continent hardest hit by the HIV epidemic. Some studies have shown that age [25–27], online behaviours [28], depression [29], education, income, social influence, and experience with mobile phone applications [27, 30] are predictors of the use of mHealth tools in connection with different health conditions in different populations and settings. However, little is known about what may predict use of mHealth solutions among members of HIV at-risk populations in sub-Saharan Africa. Identifying predictors for such use is important in understanding characteristics of individuals most likely to benefit from mHealth interventions.

The Pragmatic Trial for PrEP Roll-Out in Tanzania (PREPTA) is a research project being jointly implemented by Muhimbili University of Health and Allied Sciences (MUHAS) and University of Oslo (UiO). As part of the project, a smartphone-based application called *Jichunge* (a Swahili word that means ‘protect yourself’) was developed with the aim of supporting adherence to PrEP in two groups at higher risk of HIV infection in the Tanzanian HIV epidemic: female sex workers and men who have sex with other men. The app offers users information about PrEP, reminds them to take their daily pill, allows them to contact a doctor and/or a peer educator for online consultation services, and includes an online forum where they may engage in discussions with other PrEP users. In this paper, we evaluate the extent of and predictors for early optimal use (first 30 days of use) of the *Jichunge* app.

## Methods

### Study design and setting

This paper is part of a pragmatic quasi-experimental trial for PrEP roll-out in Tanzania (PREPTA) designed to assess the effectiveness of an mHealth intervention that aims to support adherence to PrEP among populations at higher risk of HIV in Dar es Salaam, Tanzania. Dar es Salaam is the main commercial city of Tanzania and has an estimated population of more than 4 million [31, 32]. Recent studies have estimated HIV prevalence in Dar es Salaam to be 6.3% among adult women [5] and as high as 15.3% among female sex workers [3].

### Description of the intervention

The *Jichunge* app is a smartphone application offering PrEP users information, advise, and support during start-up and use of PrEP, and its ultimate objective is to improve adherence to PrEP. A participatory design approach was adopted in the development of the app whereby members of the target population (potential end-users) were involved at different stages of the development process, details of which have been described elsewhere [33]. In the development phase, it was thought that the app had to fulfil several criteria, notably that it should provide access to quality information about PrEP, help users remember to take their daily medication, provide quick personalised feedback in case PrEP users had questions or concerns, and be perceived as attractive enough to make users maintain their use of the app over time. With the hope of fulfilling these criteria, an app was developed that offers the following functionalities:

1. The ability to set a recurrent alarm at one’s preferred PrEP-taking time every day.
2. The ability to register and keep track of one’s own pill taking, associated with a gamification element that awards users points, level upgrades and graphical rewards the longer they keep taking their PrEP regularly.
3. An editorial section providing rich information about PrEP and PrEP use.
4. An online consultation service where users can communicate with a doctor and/or a peer educator via messaging, voice call or video consultation.
5. A discussion forum where users can chat anonymously with other PrEP users.

In addition to these functions, app users can also be contacted via short message service (SMS). Some SMS communications are automated (e.g. users who have not registered pill taking for a certain number of days receive an automated text message reminder), but messages can also be sent manually (e.g. to promote new published editorial contents).

The participants do not necessarily have to have the internet access to use the Jichunge app. Some of the functions, such as the recurrent alarm that remind the user to take the daily pill, medicine registration, and normal text messages, are accessible also when the user is offline.

To protect participants’ data privacy, only consenting preregistered study participants have access to use the app, which does not store, transfer, or expose sensitive data. The app and admin portal communicates with the server using TLS, a symmetric-key encrypted communication protocol. The app allows participants to engage in chat with other app users via a function which assigns random nicknames to users and encourages them not to share person identifying information in free text. No text in the app makes explicit reference to any specific information about the project, including the populations it is targeting and the nature of the intervention. Access to the app data is limited to a few entrusted employees, requires dual factor authentication, and is continuously logged.

### Study population

The study population comprised of women aged at least 18 years who had sold sex during the 3 months immediately preceding the survey, had lived in Dar es Salaam for the past 6 months, had started PrEP treatment, and who owned a smartphone.

### Sample size estimation

The sample size was estimated using the statistical formula for cohort studies [34, 35]. Estimation assumed 50% adherence to PrEP, precision of 5%, statistical power of 80%, and a design effect of 2. After adjusting for 20% potential loss to follow-up, the minimum sample size was estimated to be 423 female sex workers.

### Sampling and recruitment

Participants were recruited between 23.03.2021 and 30.06.2021 using respondent driven sampling (RDS). RDS is a special network sampling technique that can be used to make statistical inferences about populations for which there is no existing sampling frame, such as female sex workers [35, 36]. The technique utilizes a mathematical model that weights the sample based on individual network size to mitigate the biases associated with over or under-sampling of certain groups, and thus approximates random sampling [37]. We started by recruiting three initial participants (“seeds”) who were identified during formative assessment and interviews conducted prior to the intervention. A maximum of three invitation coupons were given to each of the study participants, who were asked to use these to recruit other study participant upon completion of the interview. All participants were screened for PrEP eligibility and those who qualified were provided with PrEP pills and thereafter invited to take part in the study. Those who consented to participate received the *Jichunge* app and attended an app onboarding session before sitting for a face-to face baseline interview with a trained interviewer.

### Data collection tools and procedures

Baseline interview data were collected by trained research assistants via face-to-face interviews. While interviewing, they used a handheld tablet linked to a secure server for the storage of sensitive data (“Services for sensitive data”, or “TSD)”. TSD is highly secure server for storing and processing data with highly controlled data access and transfer [38]. The questionnaire included questions on socio-demographic characteristics, sex work, PrEP awareness, stigma, social support, alcohol use and experience with smartphones. It took about 60 minutes to complete all procedures for the study, which included screening, onboarding onto the app, and the interview. Participants were given a total of Tsh. 8000 (about USD 3.5) as compensation for transport and the time spent in the interviews and Tsh. 4000 (about USD 1.7) for each recruitee they referred to the study.

### Data on the use of the *Jichunge* app

Data on the extent of use of different *Jichunge* app functionalities (such as opening the app, registering medicine, visiting editorial contents pages, and using the online consultation services or the discussion forum) were extracted from the app administration portal.

### Variables

#### Outcome variable

The outcome variable for this analysis is *optimal use of the Jichunge app*. We define optimal users as individuals who opened the app and utilized at least three app functionalities during the first 30 days after enrolment into the study. These were users that, following a hands-on introduction to the app by a trained promoter, not only chose to open the app on their own, but also explored the app’s functionalities in a certain depth. The services of the app explored included pill registration (online or offline), visiting editorial contents, visiting the *Jichunge* app discussion forum, and consulting a peer educator or doctor via the app.

#### Independent variables

Independent variables included age, current marital status, highest education level attained, having own children, age at sex debut, age at first selling sex, steady and paying partners, condom use, income from sex work, self-reported sexually transmitted diseases, experience of physical violence, self-perceived HIV risk, self-reported health status, experience with smartphones, experience with health-related mobile phone applications, and use of common social media applications. In this study common social media applications refer to WhatsApp, Facebook, Instagram, and Twitter. We also collected information on stigma, alcohol use, PrEP awareness, and social support using the following scales:

Social support was measured using a Likert scale of 8-items, adapted from the Duke–UNC Functional Social Support Questionnaire (FSSQ) [39]. For each item, participants were asked to choose one of five responses (*1 = Much less than I would like; 2 = Less than I would like; 3 = Some, but would like more; 4 = Almost as much as I like; 5 = As much as I like*). We computed the total score for all items and a total score below 32 was considered as ‘inadequate social support’. The scale gave a Cronbach’s alpha of 0.88.

Alcohol use was measured using the Alcohol Use Disorders Identification Test (AUDIT), a questionnaire which comprises of 10 questions assessing hazardous, harmful and potentially dependency alcohol use [40, 41]. A score below 7 was defined as low risk, scores between 8 and 14 were categorised as ‘harmful’ or ‘hazardous’ use of alcohol, and above 14 was considered indicative of alcohol dependence.

PrEP awareness was measured using 8 true or false questions about PrEP. Participants who answered more than 6 questions correctly were categorized as being highly aware of PrEP.

Sex work stigma and perceived PrEP stigma were measured using 13 and 10 scale items, respectively, each with five response options (1 = Strongly disagree, 2 = Disagree, 3 = Neither disagree nor agree, 4 = Agree, 5 = Strongly agree), and gave a Cronbach alpha of 0.84 and 0.88 respectively; signifying high reliability. Sex work stigma was thereafter categorized into three groups (‘low’ for scores <=26, “moderate” for scores between 27 and 38, “high” for scores > = 39). For PrEP stigma, a score above 30 was considered “high”.

### Data analysis

Data were weighted using the individualized weight computed as the inverse proportion to the respondent’s network size. Categorical variables were summarized by using proportions, and chi-square test was used to examine the association between the outcome and independent variables. Continuous variables were summarized using median and inter-quartile range (IQR). Given that the outcome variable for this study is common (*p* = 46.4%), a modified Poisson regression model with robust standard errors (SE) was used to estimate independent predictors of optimal use of the *Jichunge* app instead of logistic regression analysis that could have over-estimated the measure of associations [42]. We first regressed each of the potential predictors with the optimal use of the app. All variables with *p* ≤ 0.2 in bivariate analysis were selected for inclusion in the multivariable Poisson regression model. Crude and adjusted prevalence ratios with corresponding 95% confidence intervals (CI) are presented. Significance value was set at 5% for all analyses. All data were analysed using STATA version 16.

## Results

### Socio-demographic characteristics and structural factors by optimal use of the *Jichunge* app

A total of 470 female sex workers were recruited, and the age of participants ranged from 18 to 50 years, with a median of 26 (IQR: 22-30) years. Weighted distributions of socio-demographics and structural factors by optimal use of the *Jichunge* app are presented in Table 1. The results show that three-quarters, 356 (75.7%) of the participants reported to have never been married, that more than half, 251 (53.4%) had attained some secondary education, and that almost two thirds, 306 (65.1%) had children.

**Table 1:** Distribution of socio-demographics and structural factors by use of the *Jichunge* app

Variable	All N (%)	Optimal users n (%)	Non-Optimal users n (%)	*p*-value^1^
**Overall**	470	218 (46.4)	252 (53.6)	
**Age**				< 0.001
18-24	218 (46.4)	84 (38.5)	134 (61.5)	
25-34	204 (43.4)	103 (50.5)	101 (49.5)	
35+	48 (10.2)	31 (64.6)	17 (35.4)	
**Marital status**				0.091
Never married	356 (75.7)	156 (43.8)	200 (56.2)	
Married or previously married	114 (24.3)	62 (54.4)	52 (45.6)	
**Education level**				0.062
No formal education	46 (9.8)	14 (30.4)	32 (69.6)	
Primary complete	173 (36.8)	77 (44.5)	96 (55.5)	
Secondary+	251 (53.4)	127 (50.6)	124 (49.4)	
**Have children**				0.446
Yes	306 (65.1)	151 (49.3)	155 (50.7)	
No	164 (34.9)	67 (40.9)	97 (59.1)	
**Financial difficulties due to spending on health**				0.018
Yes	235 (50.0)	127 (54.0)	108 (46.0)	
No	235 (50.0)	91 (38.7)	144 (61.3)	
**Sex work stigma score**				0.521
Low	33 (7.0)	12 (36.4)	21 (63.6)	
Moderate	395 (84.1)	189 (47.8)	206 (52.2)	
High	42 (8.9)	17 (40.5)	25 (69.5)	
**Reported to have had sexually transmitted disease past 6 months**				0.340
Yes	32 (6.8)	22 (68.7)	10 (31.3)	
No	437 (93.0)	196 (44.8)	241 (55.2)	
**Subjected to physical violence last 12 months**				0.095
Yes	139 (29.6)	73 (52.5)	66 (47.5)	
No	329 (70.0)	144 (43.8)	185 (56.2)	
**Self-perceived HIV risk**				0.788
High	310 (65.9)	145 (46.8)	165 (63.2)	
Medium	37 (7.9)	15 (40.5)	22 (59.5)	
Low/no	73 (15.5)	32 (43.8)	41 (56.2)	
Don’t know	46 (9.9)	24 (52.2)	22 (47.8)	
**Self-reported health status**				0.122
Very good	76 (16.2)	38 (50.0)	38 (50.0)	
Good	372 (79.1)	168 (45.2)	204 (54.8)	
Fair/poor	22 (4.7)	12 (54.5)	10 (45.5)	
**Perceived PrEP stigma**				0.985
Low	359 (76.4)	168 (46.8)	191 (53.2)	
High	111 (23.6)	50 (45.0)	61 (55.0)	
**Alcohol use (AUDIT)**				0.799
Low risk	148 (31.5)	69 (46.6)	79 (53.4)	
Harmful or hazardous	137 (29.1)	67 (48.9)	70 (51.1)	
Alcohol dependence	185 (39.4)	82 (44.3)	103 (55.7)	
**PrEP awareness**				< 0.001
Low	254 (54.0)	96 (37.8)	158 (62.2)	
High	216 (46.0)	122 (56.5)	94 (43.5)	
**Social support**				0.153
Inadequate	290 (61.7)	133 (45.9)	157 (54.1)	
adequate	175 (37.2)	82 (46.9)	93 (53.1)	

^1^*p*-value is based on the chi-square test

Almost half, 218 (46.4%) of the participants were optimal users of the *Jichunge* app. Optimal use was significantly associated with higher age (*p* <  0.001), reported financial difficulties due to spending on health (*p* = 0.018) and high PrEP awareness (*p* <  0.001) (Table 1).

### Sex work characteristics by optimal use of the *Jichunge* app

The median age at sex debut and at first selling of sex was 17 (IQR: 15-18) years and 20 (IQR: 18-22) years, respectively. About half, 232 (49.4%) of the participants had used a condom during the most recent sexual encounter with a paying partner, and 220 (46.8%) said that they sometimes accepted condom-less sex for increased payment. Optimal use of the *Jichunge* app was associated with age at sex debut (*p* = 0.033) and marginally associated with income from sex work (*p* = 0.055) (Table 2).

**Table 2:** Distribution of sex work characteristics by optimal use of the *Jichunge* app

Variable	All N (%)	Optimal users n (%)	*p*-value^1^
**Age at sex debut (years)**			0.033
Below 15	65 (13.8)	22 (33.8)	
15-17	193 (41.1)	105 (54.4)	
18+	212 (45.1)	91 (42.9)	
**Age at first selling of sex (years)**			0.389
Below 18	88 (18.7)	34 (38.6)	
18-24	290 (61.7)	136 (46.9)	
25+	92 (19.6)	48 (52.2)	
**Reported monthly earnings from sex work (TZS**^**2**^**)**			0.055
≤ 150,000	124 (26.4)	53 (42.7)	
150,001-299,999	93 (19.8)	43 (46.2)	
300,000-444,999	149 (31.7)	85 (57.0)	
≥ 450,000	89 (18.9)	29 (32.6)	
**Amount paid by last client (TZS**^**2**^**)**			0.266
≤ 15,000	125 (26.6)	59 (47.2)	
15,001-25,000	118 (25.1)	50 (42.4)	
25,001-39,999	85 (18.1)	38 (44.7)	
≥ 40,000	142 (30.2)	71 (50.0)	
**Having a steady partner**			0.740
Yes	302 (64.3)	144 (47.7)	
No	168 (35.7)	74 (44.0)	
**Number of sexual partners last months**			0.380
< 10	158 (33.6)	67 (42.4)	
10-29	165 (35.1)	74 (44.8)	
≥ 30	147 (31.3)	77 (52.4)	
**Used condom during last sex with paying partner**			0.433
Yes	232 (49.4)	115 (49.6)	
No	238 (50.6)	103 (43.3)	
**Accepting condom-less sex for more pay**			0.340
Yes	220 (46.8)	108 (49.1)	
No	250 (53.2)	110 (44.0)	

^1^*p*-value is based on the chi-square test; ^2^TZS stands for Tanzanian Shillings (1 USD =2318 TZS)

### Smartphone experiences by optimal use of the *Jichunge* app

While more than half, 275 (58.5%) of the participants reported to be familiar with the use of smartphones, almost all, 465 (98.9%) reported to never have used a health-related mobile phone application in the past. A large proportion of the participants, 335 (71.3%) reported to have used three or more of the common, mainstream social media applications. More specifically, 442 (94%) reported having used WhatsApp, 450 (95.7%) Facebook, and 352 (74.9%) Instagram, whereas only 20 (4.3%) reported having used Twitter. The findings further show that optimal use of the *Jichunge* app was significantly associated with using several (at least three) of the common social media apps (*p* = 0.003) (Table 3).

**Table 3:** Smartphone experience by optimal use of *Jichunge* app

Variable	All N (%)	Optimal users n (%)	*p*-value^1^
**Familiarity with a smartphone**			0.786
Very familiar	190 (40.4)	96 (50.5)	
Familiar	275 (58.5)	119 (43.3)	
Unfamiliar	5 (1.1)	3 (60.0)	
**Frequency of opening data in the smartphone**			0.905
At least once per day	383 (81.5)	181 (47.3)	
Less than once per day	87 (53.0)	37 (42.5)	
**Amount spent on internet bundles in the past week (TZS**^**2**^**)**			0.580
< 5000	221 (47.5)	108 (48.9)	
5000-9999	166 (35.7)	76 (45.8)	
10,000+	78 (16.8)	34 (43.6)	
**Ever having used health related mobile phone apps**			0.079
Yes	5 (1.1)	3 (60.0)	
No	465 (98.9)	215 (46.2)	
**Having used phone for health information in past month**			0.355
Yes	24 (51.4)	12 (50.0)	
No	443 (94.9)	203 (45.8)	
**Use of social media applications**			
WhatsApp	442 (94.0)	212 (48.0)	0.052
Facebook	450 (95.7)	206 (45.8)	0.413
Instagram	352 (74.9)	181 (51.4)	0.001
Twitter	20 (4.3)	11 (55.0)	0.078
Several (3 or more apps)	335 (71.3)	171 (51.0)	0.003

^1^*p*-value is based on the chi-square test; ^2^TZS stands for Tanzanian Shillings (1 USD =2318 TZS)

### Use of different *Jichunge* app services

**Fig. 1:**
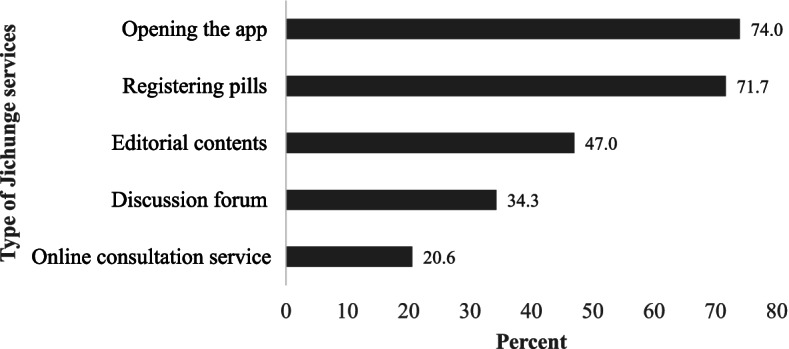
Distribution of participants use of different *Jichunge* app services during the first 30 days of app ownership

Three quarters of participants (74.0%) had opened the app and 71.7% had registered their pill taking at least once in their first 30 days as *Jichunge* app users. Nearly half (47.0%) of the participants had visited the editorial contents, where different information about PrEP can be accessed. The discussion forum and the online consultation service with a doctor or a peer educator was used by 34.3 and 20.6% of participants, respectively (Fig. 1).

### Independent predictors of optimal use of the *Jichunge* app

In Table 4, we present results from the multivariable Poisson regression model for the predictors of optimal use of the *Jichunge* app. Participants aged 25-34 years (aPR = 1.3; 95% CI: 1.10-1.65, *p* = 0.004) and those aged 35 years and above (aPR = 1.6; 95% CI: 1.19-2.07, *p* = 0.001) had 30 and 60% higher prevalence of optimal use of the app than those aged 18-24 years. Moreover, participants having at least secondary education had 80% higher prevalence of optimal use of the app when compared to those with no formal education (aPR = 1.8; 95% CI: 1.08-2.94, *p* = 0.023). Optimal use was also significantly associated with self-perceived inadequate social support (aPR = 1.2; 95% CI: 1.02-1.48, *p* = 0.030), having high PrEP awareness (aPR = 1.3; 95% CI: 1.08-1.55, *p* = 0.005), and using common social media apps (aPR = 1.4; 95% CI: 1.08-1.71, *p* = 0.009).

**Table 4:** Modified Poisson regression modelling of independent predictors of optimal use of the *Jichunge* app

Variable	Crude estimates		Adjusted estimates	
	PR (95% CI)	***p***-value	aPR (95% CI)	***p***-value
**Age**				
18-24	Ref.	Ref.	Ref.	Ref.
25-34	1.4 (1.14-1.69)	0.001	1.3 (1.10-1.65)	0.004
35+	1.8 (1.38-2.24)	< 0.001	1.6 (1.19-2.07)	0.001
**Marital status**				
Never married	Ref.	Ref.	Ref.	Ref.
Married or previously married	1.2 (0.46-0.56)	0.076	1.1 (0.91-1.35)	0.311
**Education level**				
No formal education	Ref.	Ref.	Ref.	Ref.
Primary	1.6 (0.92-2.80)	0.098	1.6 (0.98-2.69)	0.061
Secondary+	1.7 (0.99-2.98)	0.053	1.8 (1.08-2.94)	0.023
**Income from sex work**				
≤ 150,000	Ref.	Ref.	Ref.	Ref.
150,001-299,999	1.1 (0.82-1.36)	0.678	1.0 (0.81-1.33)	0.763
300,000-444,999	1.2 (0.98-1.53)	0.079	1.2 (0.95-1.47)	0.132
≥ 450,000	0.87 (0.64-0.60)	0.347	0.80 (0.67-1.20)	0.472
**Financial difficulties due to spending on health**				
Yes	1.2 (1.03-1.47)	0.020	1.1 (0.95-1.35)	0.173
No	Ref.	Ref.	Ref.	Ref.
**Age at sex debut (years)**				
Below 15	Ref.	Ref	Ref.	Ref
15-17	1.4 (1.01-1.88)	0.038	1.3 (0.98-1.77)	0.065
18+	1.2 (0.84-1.60)	0.372	0.99 (0.72-1.36)	0.957
**Subjected to physical violence past 12 months**				
Yes	1.2 (0.98-1.38)	0.091	1.0 (0.88-1.25)	0.591
No	Ref.	Ref.	Ref.	Ref.
**Social support**				
Inadequate	1.1 (0.95-1.37)	0.161	1.2 (1.02-1.48)	0.030
Adequate	Ref.	Ref.	Ref.	Ref.
**Self-reported health status**				
Very good	Ref.	Ref.	Ref.	Ref.
Good	0.86 (0.70-1.07)	0.177	0.96 (0.78-1.19)	0.705
Fair/poor	1.1 (0.84-1.57)	0.386	1.0 (0.72-1.42)	0.944
**PrEP awareness**				
High	1.5 (1.24-1.77)	< 0.001	1.3 (1.08-1.55)	0.005
Low	Ref.	Ref.	Ref.	Ref.
**Ever used health a related mobile phone application**				
Yes	0.87 (0.73-1.04)	0.136	1.2 (0.92-1.68)	0.160
No	Ref.	Ref.	Ref.	Ref.
**Use of common social media apps**				
Yes	1.4 (1.10-1.77)	0.006	1.4 (1.08-1.71)	0.009
No	Ref.	Ref.	Ref.	Ref.

*PR* Prevalence ratio, *aPR* Adjusted prevalence ratio

## Discussion

mHealth has been shown to be a promising tool in promoting adherence to PrEP [20]. In this study, we aimed to determine the extent of and the predictors for optimal use of a smartphone-based application aiming to support adherence to PrEP among female sex workers in Dar es Salaam, Tanzania. The findings we report might provide useful inputs during the implementation of the Tanzania Digital Health Strategy 2019-2024 [43] and the Health Sector Strategic Plan 2021-2026 [44] which outline strategies for leveraging digital health technology to facilitate the achievement of universal health coverage. The study may also be used to inform the implementation of the 2016 Tanzania National Policy Guidelines for Collaborative TB/HIV Activities [45] and Tanzania National Guideline for Comprehensive Package of HIV Interventions for Key and Vulnerable Populations [46], which stress on identifying and recommending specific interventions targeting people at increased risk of HIV transmission.

Overall, nearly half of the participants optimally used the *Jichunge* app. Being older (above 24 years), having at least secondary education, demonstrating high awareness of PrEP, experiencing a relative lack of social support, and having experience with common mainstream social media apps, significantly predicted the optimal use of the app. That a large proportion of participants used different mHealth functionalities, such as pill registration, editorial contents, and online consultation with a doctor or a peer educator, is an indication of acceptability of the *Jichunge* app. The findings are in line with a study of the role of mHealth in promoting adherence to PrEP among young people at increased risk of HIV in United States, which demonstrated high acceptability of mHealth [21]. A recent systematic review on mHealth targeting behavioural change indicated that while many applications have been developed and in use, there is limited evaluation of their usability [47]. This paper therefore presents critical information that may inform future use and modification of *Jichunge* app to enhance its effectiveness.

While young people have been reported to be more likely to use mHealth due to their technological skills [27], our findings from this sex worker population indicate that persons of older age were more likely to use the app. This could be because older female sex workers may have better understood the HIV-related risks associated with the selling of sex and hence were more determined to protect themselves than their younger counterparts. More dedicated use among person in older age groups have previously been observed in a study of an mHealth application for the assessment and prevention of heart disease [25], and higher age was also found to be a predictor for long-term use of an mHealth intervention for diabetes management [26]. These findings may highlight a need to provide more motivation for your people to use mHealth apps by tailoring their design and functionality to suit the needs and preferences of adolescents and young adults.

Use of the *Jichunge* app was independently associated with higher education level. This corroborate findings from other studies which have found high literacy to be associated with awareness and use of various mHealth tools [48–50]. The *Jichunge* app, like many other mHealth apps, may be more attractive to people who are good at reading, understanding and internalizing health information. Our findings are also consistent with the study by Khatume et al. [27] in the United States which found that individuals with higher education were more likely to use mHealth apps of different types than those with a lower education level. Inclusion of features such as audios or videos that can attract people with lower reading skills may possibly help attract more users to mHealth apps.

Female sex workers who demonstrated high PrEP awareness were significantly more likely to optimally use the *Jichunge* app. A systematic review on PrEP awareness and use in low and middle income countries have indicated that PrEP awareness and knowledge to be associated with PrEP acceptability and use [51]. Thus, people with prior exposure to PrEP information may have been more motivated to use PrEP and hence more interested in learning about PrEP through the *Jichunge* app. Similar findings have been reported from the PrEP4Love study, which examined the role of text messaging in promoting PrEP uptake [52]. This suggests that interventions to raise health awareness, in our case PrEP awareness, may be key in facilitating the use of mHealth solutions. Combining mHealth interventions with different other types of programming (such as PrEP awareness campaigns) may be more likely to be effective than mHealth solutions on their own.

This study found that participants with lower self-perceived social support were more likely to be optimal users of the *Jichunge* app than persons who perceived their social support to be adequate. Digital technologies have increasingly affected human interaction and there may be an inclination to replace more direct human social interaction with digitally mediated interaction [53]. A relative lack of social support could perhaps subconsciously have prompted users to embrace a digital solution more extensively such as the *Jichunge* app as a supplement to direct social interaction. Notably, research from elsewhere has found that some individuals consider mHealth tools as additional support for different behaviours and aspects of life [20, 21]. Particularly the more personally interactive elements of the *Jichunge* app (the discussion forum and the online consultation services) may perhaps have attracted users with inadequate social support. Our findings suggest that there may be a strong rationale for incorporating some types of social interaction and support services in mHealth tools.

Experience with use of mainstream social media apps was associated with optimal use of the *Jichunge* app. Having used social media applications may mean that a person is electronically savvy and spends more time online and this may increase the likelihood of using mHealth internet-based interventions as proposed in models for online behaviour interventions [54, 55]. This observation is in line with our earlier mentioned findings that high literacy and PrEP awareness were associated with optimal use of the *Jichunge* app. Finally, learning from mainstream social media apps, that have been developed with considerably more resources than most mHealth apps, may be critical in designing applications which attract users and promote effective use for intended behavioural change.

### Limitations

This study has some limitations: First, participants needed to have a smartphone and they may hence not represent the female sex workers population in the study area since not all of them own a smartphone. Future studies should consider evaluating the mHealth apps which can work in any mobile device. Second, the definition of optimal use of the app did not take into consideration the frequency of use of different features of the app. While this may perhaps have led to overestimation of optimal use of mHealth, it may have had little effect on the predictors for optimal use of the app, which is the central theme in this paper. Third, use was measured over a short period, and further studies should assess predictors of more long-term engagement with mHealth interventions among Female sex workers. Fourth, the study had a cross-sectional design, which limits the ability to draw conclusions about causal relationships between variables. Thus, there is a need for longitudinal studies to establish casual relationships.

## Conclusion

mHealth interventions aiming to support start-up and use of PrEP among female sex workers is feasible and promising. A substantial number of female sex workers in Dar es Salam, Tanzania demonstrated optimal use of the *Jichunge* app. Optimal use was associated with older age, higher education level, higher awareness of PrEP, lower social support, and more extensive experience with common mainstream social media applications. The findings from this study may serve as the guide for mHealth development and public health interventions in understanding individuals most likely to benefit from mHealth services.

## Data Availability

The data for this analysis will be available from the principal investigator (PI) of the PREPTA project upon reasonable request. Name of PI: Prof. Elia J Mmbaga; Email: elia.mmbaga@medisin.uio.no

## Abbreviations

ART: Antiretroviral Therapy
HIV: Human Immunodeficiency Virus
mHealth: Mobile Health
PrEP: Pre-Exposure Prophylaxis
PREPTA: Pragmatic Trial for HIV Pre-Exposure Prophylaxis Roll-Out in Tanzania
RDS: Respondent Driven Sampling

## References

[1] Shannon K, Crago AL, Baral SD, Bekker LG, Kerrigan D, Decker MR (2018). The global response and unmet actions for HIV and sex workers. Lancet.

[2] Baral S, Beyrer C, Muessig K, Poteat T, Wirtz AL, Decker MR (2012). Burden of HIV among female sex workers in low-income and middle-income countries: a systematic review and meta-analysis. Lancet Infect Dis.

[3] Mizinduko MM, Moen K, Likindikoki S, Mwijage A, Leyna GH, Makyao N (2020). HIV prevalence and associated risk factors among female sex workers in Dar Es Salaam, Tanzania: tracking the epidemic. Int J STD AIDS.

[4] National AIDS Control Program (2013). Tanzania (2013): HIV biological and behavioral surveys among female sex workers in seven regions. Round [1]. - PSI/Tanzania Dataverse.

[5] Tanzania Commission for AIDS (TACAIDS) and Zanzibar AIDS Commission (ZAC. The Tanzania HIV Impact Survey 2016-2017 (THIS) - Final Report. 2018. Available from: https://www.nbs.go.tz/index.php/en/census-surveys/health-statistics/hiv-and-malaria-survey/382-the-tanzania-hiv-impact-survey-2016-2017-this-final-report. [cited 2022 Jan 16].

[6] WHO (2019). HIV prevention, diagnosis, treatment and care for key populations policy brief consolidated guidelines.

[7] Pre-Exposure Prophylaxis (PrEP) | HIV Risk and Prevention | HIV/AIDS | CDC. Available from: https://www.cdc.gov/hiv/risk/prep/index.html. [cited 2020 Jan 13].

[8] Jiang J, Yang X, Ye L, Zhou B, Ning C, Huang J (2014). Pre-exposure prophylaxis for the prevention of HIV infection in high risk populations: a meta-analysis of randomized controlled trials. Kissinger P, editor. PLoS One.

[9] McCormack S, Dunn DT, Desai M, Dolling DI, Gafos M, Gilson R (2016). Pre-exposure prophylaxis to prevent the acquisition of HIV-1 infection (PROUD): effectiveness results from the pilot phase of a pragmatic open-label randomised trial. Lancet.

[10] Marrazzo JM, Ramjee G, Richardson BA, Gomez K, Mgodi N, Nair G (2015). Tenofovir-based Preexposure prophylaxis for HIV infection among African Women. N Engl J Med.

[11] Cowan FM, Delany-Moretlwe S (2016). Promise and pitfalls of pre-exposure prophylaxis for female sex workers. Curr Opin HIV AIDS.

[12] Baeten JM, Haberer JE, Liu AY, Sista N (2013). Preexposure prophylaxis for HIV prevention: where have we been and where are we going?. J Acquir Immune Defic Syndr.

[13] Moore DJ, Jain S, Dubé MP, Daar ES, Sun X, Young J (2018). Randomized controlled trial of daily text messages to support adherence to Preexposure prophylaxis in individuals at risk for human immunodeficiency virus: the TAPIR study. Clin Infect Dis.

[14] World Health Organization. Global difusion of eHealth: making universal health coverage achievable. Report of the third global survey on eHealth. Geneva: World Health Organization; 2016. https://apps.who.int/iris/handle/10665/252529.

[15] Catalani C, Philbrick W, Fraser H, Mechael P, Israelski DM (2013). mHealth for HIV treatment & prevention: a systematic review of the literature. Open AIDS J.

[16] Beratarrechea A, Lee AG, Willner JM, Jahangir E, Ciapponi A, Rubinstein A (2014). The impact of mobile health interventions on chronic disease outcomes in developing countries: a systematic review. Telemed J E Health.

[17] Horvath T, Azman H, Kennedy GE, Rutherford GW. Mobile phone text messaging for promoting adherence to antiretroviral therapy in patients with HIV infection. Vol. 2017, cochrane database of systematic reviews. Hoboken: Wiley Ltd; 2012. 10.1002/14651858.CD009756 PMC6486190 22419345

[18] Hardy H, Kumar V, Doros G, Farmer E, Drainoni ML, Rybin D (2011). Randomized controlled trial of a personalized cellular phone reminder system to enhance adherence to antiretroviral therapy. AIDS Patient Care STDs.

[19] Mbuagbaw L, Mursleen S, Lytvyn L, Smieja M, Dolovich L, Thabane L. Mobile phone text messaging interventions for HIV and other chronic diseases: an overview of systematic reviews and framework for evidence transfer. Vol. 15, BMC health services research: BioMed Central Ltd. London: Springer Nature; 2015. 10.1186/s12913-014-0654-6 PMC4308847 25609559

[20] Fuchs JD, Stojanovski K, Vittinghoff E, McMahan VM, Hosek SG, Amico KR (2018). A Mobile health strategy to support adherence to antiretroviral Preexposure prophylaxis. AIDS Patient Care STDs.

[21] Liu AY, Vittinghoff E, von Felten P, Rivet Amico K, Anderson PL, Lester R (2019). Randomized controlled trial of a Mobile health intervention to promote retention and adherence to Preexposure prophylaxis among Young people at risk for human immunodeficiency virus: the EPIC study. Clin Infect Dis.

[22] Pintye J, Rogers Z, Kinuthia J, Mugwanya KK, Abuna F, Lagat H (2020). Two-way Short message service (SMS) communication may increase pre-exposure prophylaxis continuation and adherence among pregnant and postpartum women in Kenya. Glob Heal Sci Pract.

[23] Weitzman PF, Zhou Y, Kogelman L, Rodarte S, Vicente SR, Levkoff SE (2021). mHealth for pre-exposure prophylaxis adherence by young adult men who have sex with men. mHealth.

[24] Ngugi EN, Roth E, Mastin T, Nderitu MG, Yasmin S (2012). Female sex workers in Africa: epidemiology overview, data gaps, ways forward.

[25] Goyal S, Morita PP, Picton P, Seto E, Zbib A, Cafazzo JA (2016). Uptake of a consumer-focused mHealth application for the assessment and prevention of heart disease: the <30 days study. JMIR MHealth UHealth.

[26] Garabedian LF, Ross-Degnan D, LeCates RF, Wharam JF (2019). Uptake and use of a diabetes management program with a mobile glucometer. Prim Care Diabetes.

[27] Carroll JK, Moorhead A, Bond R, LeBlanc WG, Petrella RJ, Fiscella K (2017). Who uses mobile phone health apps and does use matter? A secondary data analytics approach. J Med Internet Res.

[28] Russell TG, Gillespie N, Hartley N, Theodoros D, Hill A, Gray L (2015). Exploring the predictors of home telehealth uptake by elderly Australian healthcare consumers. J Telemed Telecare.

[29] Psihogios AM, King-Dowling S, O’Hagan B, Darabos K, Maurer L, Young J (2021). Contextual predictors of engagement in a tailored mHealth intervention for adolescent and Young adult Cancer survivors. Ann Behav Med.

[30] Cho J, Kim S (2020). Personal and social predictors of use and non-use of fitness/diet app: application of random Forest algorithm. Telematics Inform.

[31] National Bureau of Statistics (2013). The United Republic of Tanzania 2012 population and housing census: population distribution by administrative areas national bureau of statistics ministry of finance Dar es Salaam.

[32] National Bureau of Statistics. United Republic of Tanzania: National population projections. United Republic of Tanzania; 2018. Available from: www.nbs.go.tz. [cited 2022 Jan 16]

[33] Mauka W, Mbotwa C, Moen K, Lichtwarck HO, Haaland I, Kazaura M (2021). Development of a mobile health application for hiv prevention among at-risk populations in urban settings in east africa: a participatory design approach. JMIR Form Res.

[34] Kirkwood BR, JAC S. Essential medical statistics. 2nd ed. Hoboken: Blackwell Publishing Company; 2003.

[35] World Health Organization (WHO) (2013). Introduction to HIV/AIDS and sexually transmitted infection surveillance module 4: introduction to respondent-driven sampling. Introduction to HIV/AIDS and sexually transmitted infection surveillance.

[36] Heckathorn DD (1997). Respondent-driven sampling: a new approach to the study of hidden populations. Soc Probl.

[37] Schonlau M, Liebau E, Berlin D (2012). Respondent-driven sampling. Stata J.

[38] UiO. About TSD. Available from: https://www.uio.no/english/services/it/research/sensitive-data/about/description-of-the-system.html. [cited 2022 Feb 26].

[39] Broadhead WE, Gehlbach SH, De Gruy FV, Kaplan BH (1988). The Duke–UNC functional social support questionnaire: measurement of social support in family medicine patients. Med Care.

[40] Saunders JB, Og A, TF GB, De La Fuente JR, Grant M (1993). Development of the alcohol use disorders identification test (AUDIT): WHO collaborative project on early detection of persons with harmful alcohol consumption-II. Addiction.

[41] Vissoci JRN, Hertz J, El-Gabri D, Andrade Do Nascimento JR, Pestillo De Oliveira L, Mmbaga BT (2018). Cross-cultural adaptation and psychometric properties of the AUDIT and CAGE questionnaires in Tanzanian Swahili for a traumatic brain injury population. Alcohol Alcohol.

[42] Yelland LN, Salter AB, Ryan P (2011). Performance of the modified Poisson regression approach for estimating relative risks from clustered prospective data. Am J Epidemiol.

[43] United Republic of Tanzania (2021). Ministry of health community development gender elderly and children. digital health strategy July 2019 - June 2024.

[44] United Republic of Tanzania (2021). Ministry of health community development gender elderly and children. Health sector strategic plan 2021-2026.

[45] United Republic of Tanzania (2016). Ministry of health and social welfrare. National policy guidelines for collaborative TB/HIV activities.

[46] United Republic of Tanzania (2017). Ministry of health community development gender elderly and children. National AIDS control program. National guideline for comprehensive package of HIV interventions for key and vulnerable populations.

[47] McKay FH, Cheng C, Wright A, Shill J, Stephens H, Uccellini M (2016). Evaluating mobile phone applications for health behaviour change: a systematic review. J Telemed Telecare.

[48] Ramachandran N, Srinivasan M, Thekkur P, Johnson P, Chinnakali P, Naik BN (2015). Mobile phone usage and willingness to receive health-related information among patients attending a chronic disease Clinic in Rural Puducherry. India J Diabetes Sci Technol.

[49] Schrauben SJ, Appel L, Rivera E, Lora CM, Lash JP, Chen J (2021). Mobile health (mHealth) technology: assessment of availability, acceptability, and use in CKD. Am J kidney Dis Off J Natl Kidney Found.

[50] Zhou J, Wang C (2020). Improving cancer survivors’ e-health literacy via online health communities (OHCs): a social support perspective. J Cancer Surviv.

[51] Yi S, Tuot S, Mwai GW, Ngin C, Chhim K, Pal K (2017). Awareness and willingness to use HIV pre-exposure prophylaxis among men who have sex with men in low- and middle-income countries: a systematic review and meta-analysis. J Int AIDS Soc.

[52] Phillips GII, Raman AB, Felt D, McCuskey DJ, Hayford CS, Pickett J, et al. PrEP4Love: the role of messaging and prevention advocacy in PrEP attitudes, perceptions, and uptake among YMSM and transgender women. JAIDS J Acquir Immune Defic Syndr. 2020;83(5) Available from: https://journals.lww.com/jaids/Fulltext/2020/04150/PrEP4Love__The_Role_of_Messaging_and_Prevention.2.aspx. 10.1097/QAI.0000000000002297 PMC7083076 31939870

[53] Feldman DI, Theodore Robison W, Pacor JM, Caddell LC, Feldman EB, Deitz RL (2018). Harnessing mHealth technologies to increase physical activity and prevent cardiovascular disease. Clin Cardiol.

[54] Ritterband LM, Thorndike FP, Cox DJ, Kovatchev BP, Gonder-Frederick LA (2009). A behavior change model for internet interventions. Ann Behav Med.

[55] Short CE, Rebar AL, Ronald C (2016). Diseñar intervenciones de cambio de comportamiento en línea atractivas: un modelo propuesto de participación del usuario. Eur Heal Psychol.

